# Gluten-Free Flatbread with Carob Flour and Sourdough: Nutritional Composition, Technological Properties and Storage Stability

**DOI:** 10.3390/foods15091504

**Published:** 2026-04-25

**Authors:** Bojana Voučko, Saša Drakula, Nikolina Čukelj Mustač, Vedrana Pleš, Ljiljana Nanjara, Tomislava Grgić, Dubravka Novotni

**Affiliations:** 1 Department of Food Engineering, Faculty of Food Technology and Biotechnology, University of Zagreb, 10 000 Zagreb, Croatia; 2 Department of Food Processing Technology, Karlovac University of Applied Sciences, 47 000 Karlovac, Croatia; 3 Department of Food Technology, University of Applied Sciences “Marko Marulić”, 22 300 Knin, Croatia

**Keywords:** carob seeds, gluten-free flatbread, sensory analysis, texture profile analysis, whole carob fruit

## Abstract

The growing demand for clean-label foods has stimulated interest in minimally processed ingredients capable of improving the nutritional and technological quality of gluten-free bakery products. Carob (*Ceratonia siliqua* L.) is an underutilized Mediterranean crop whose seeds are mainly used for locust bean gum production, while other fractions of the fruit remain insufficiently valorized. This study investigated the potential of carob seed flour (CSF) and the whole carob fruit flour (pods and seeds; CSPF) as natural structuring ingredients in gluten-free flatbread (GFFB), combined with sourdough fermentation. The initial technological properties (pasting profile, baking loss, specific volume, color, and texture profile) and nutritional composition were evaluated, alongside storage stability, through textural and sensory changes during 72 h. The incorporation of carob and millet ingredients improved the nutritional profile of GFFB, increasing total dietary fiber by over 60% and nearly doubling the iron content, without compromising sensory acceptance. CSF use resulted in an improved pasting profile and a 50% softer crumb structure. Sourdough fermentation successfully mitigated the increased hardness and lower sensory freshness perception in CSPF formulations. Carob seed flour, as well as whole carob fruit flour combined with sourdough, represent effective natural strategies for improving the technological properties, nutritional quality, texture profile, and freshness perception of gluten-free flatbread without compromising sensory acceptability.

## 1. Introduction

Despite recent advancements, many commercially available gluten-free bakery products maintain a nutritional gap in protein and fiber content compared to their gluten-containing counterparts. Thus, incorporating whole-grain alternative flours and pseudocereals represents a suitable option for improving their nutritional and sensory quality [1,2]. Efforts to improve the nutritional value of gluten-free bread may result in the deterioration of texture, sensory quality, or shelf life [3]. The absence of gluten, the key protein responsible for forming the viscoelastic network in wheat dough, limits the development of a stable crumb structure in gluten-free bread [4,5]. To compensate for these drawbacks, hydrocolloids, proteins, fats, emulsifiers, and other structuring agents are commonly used as technological improvers [6]. However, the inclusion of multiple structuring ingredients often creates the perception of a highly processed product [6]. Despite their technological benefits, the use of chemical additives (e.g., hydrocolloids) in food products is increasingly questioned due to growing consumer demand for more natural and minimally processed “clean-label” foods [7,8]. Replacing these additives with minimally processed, whole-ingredient alternatives, while maintaining desirable technological and sensory properties, remains a significant challenge. This is particularly evident in the development of nutritionally improved gluten-free bread, where the possibility of utilizing minimally processed ingredients as functional alternatives to complex additives is still being explored. Incorporation of alternative flours and the application of bioprocessing technologies have been shown to be promising strategies to improve both the nutritional and technological quality of gluten-free bakery products [9].

The Mediterranean region accounts for more than 90% of the global production of carob (*Ceratonia siliqua* L.). However, the utilization of this crop remains suboptimal. The carob fruit consists of the pod (approximately 90%) and the seed (approximately 10%) [10]. Carob seeds are widely used in the food industry for the production of locust bean gum, a thickening and gelling additive (E410) derived from the endosperm, which represents 42–46% of the seed mass, while the remaining fractions remain underutilized [10,11]. In addition to its thickening properties, comparable to those of synthetic hydrocolloids, locus bean gum has been reported to attenuate postprandial serum glucose responses by slowing the rate of food passage through the digestive tract [12], as well as to exhibit prebiotic potential [13]. Nevertheless, the application of carob as a functional ingredient of gluten-free formulations has been only sparsely investigated. Fractions of the carob seed flour were successfully used in improving gluten-free bread hardness and porosity [14]. Several studies have used carob gum extracts or purified LBG to improve the texture and sensory quality of gluten-free bread [15,16]. While data on carob pulp powder in gluten-free bread are limited, studies in wheat dough have shown that its introduction can cause a major increase in wheat dough resistance while significantly decreasing extensibility. These rheological changes typically result in a lower bread volume and a denser crumb structure. Specifically, at substitution levels of 20%, carob can negatively impact the product’s taste, leading to an undesirable doughy flavor and smearing in the mouth during chewing [17]. Nevertheless, the use of purified hydrocolloids or isolated extracts may increase the cost of already expensive gluten-free products and does not fully exploit the potential of the whole carob fruit, which naturally contains dietary fiber, proteins, polyphenols, and other bioactive compounds with nutritional and technological functionality [11]. Furthermore, amongst the studies that have focused on gluten-free bread, considerably less attention has been given to gluten-free flatbread (GFFB). Flatbreads are widely consumed bakery products characterized by their relatively thin structure and simple processing, which makes them particularly suitable for the incorporation of alternative ingredients such as pseudocereals or legumes [18]. Even fewer studies have investigated the potential of carob sourdough in either gluten-containing or gluten-free breadmaking, with only a limited number of reports available [2,9,19,20]. To the best of our knowledge, none of these studies have examined the influence of carob-based sourdough on the short-term storage stability of gluten-free products as assessed through textural and sensory changes. Building on previous work [9], this study expands current knowledge by comparing different levels of carob utilization, including flour obtained from the whole carob fruit (pod and seed; CSPF), thereby promoting the valorization of the entire carob fruit, carob seed flour (CSF) used as a whole ingredient rather than as an isolated hydrocolloid, and sourdough-fermented mixtures of CSPF. The research evaluates the impact of different carob flours (CSPF and CSF) on pasting properties of flour blends, as well as the nutritional, technological, and sensory quality of GFFB during 72 h of storage. Additionally, the impact of sourdough fermentation on the storage properties and quality of CSPF GFFB was assessed.

## 2. Materials and Methods

### 2.1. Ingredients and Starter Cultures

Whole grain rice flour (RF; Nutrigold, Bulgaria, distributed by Galleria Internazionale, Zagreb, Croatia), white corn flour (WCF; Danijel Sinković, family-run farm, Bedekovčina, Croatia), proso millet flour (PMF; BEZGLUTEN, Posądza, Poland), proso millet bran (PMB, Mlinopek l.c., Murska Sobota, Slovenia), whole carob fruit flour (CSPF; Šafran Inc., Zagreb, Croatia), and carob seed flour (CSF; family-run farm, Šipan, Croatia) were the main ingredients. Other raw materials used to prepare bread included tap water, fine sea salt (Solana Pag, Pag, Croatia), granulated white sugar (Viro, Virovitica, Croatia), edible sodium bicarbonate (Franck Inc., Zagreb, Croatia), refined sunflower oil (Zvijezda Ltd., Zagreb, Croatia), active dry instant yeast (Lesaffre Adriatic, Prigorje Brdovečko, Croatia), and vegetable-based spray oil for baking (Trennaktiv PR 100, DÜBÖR Groneweg GmbH, Germany). *Limosilactobacillus fermentum* (LF) (DSM 20052) was provided by Deutsche Sammlung von Mikroorganismen and Zellkulturen (DSMZ, Braunschweig, Germany), while *Kluyveromyces marxianus* (KM) (NBRC 1777) was donated by the Laboratory for Biochemical Engineering, Industrial Microbiology and Malting and Brewing Technology, University of Zagreb Faculty of Food Technology and Biotechnology (UNZG FFTB, Zagreb, Croatia). The chemical composition of raw materials has been previously reported [9].

### 2.2. Bread Samples

The control bread (CTRL-B) was formulated solely from RF and WCF flour, whereas CSF-B and CSPF-B contained the flour mixture consisting of PMF and PMB with the respective carob ingredient CSF or CSPF (Table 1). Further on, the CSPF-based formulation was made both without sourdough (CSPF-B) and with sourdough (CSPF_LF+KM), following the optimized sourdough protocol previously established [9].

### 2.3. Measurement of Pasting Properties

Pasting properties were determined for the individual ingredients and for the flour blends used in the bread formulations presented in Table 1, without sourdough addition. Samples were analyzed in a Micro Visco-Amylo-Graph (Brabender GmbH & Co. KG, Duisburg, Germany) with ramped temperature and stirring at variable shearing speeds following a modified ICC standard 162 [21]. Samples of total weight of 10 g were mixed with distilled water in a canister (corr. 14% solids, *w*/*w*). Suspensions were equilibrated at 25 °C, heated to 50 °C at a rate of 12.5 °C min^−1^, and held for 1 min, heated to 95 °C at a rate of 12.5 °C min^−1^, held at 95 °C for 2.5 min, cooled to 50 °C at a rate of 12.5 °C min^−1^, and held at 50 °C for 1 min. A constant paddle rotating speed (250 rpm) and a measuring range of 150 cmg were used throughout the entire analysis for all test measurements.

### 2.4. Breadmaking Procedure

Bread was prepared using an indirect method, involving the preliminary preparation of scalded white corn flour (ScD) and, where applicable, sourdough fermentation. WCF was scalded with 110% of boiling tap water (flour basis) in a plastic container, mixed for 3 min (Robert Bosch GmbH, Gerlingen, Germany), cooled to room temperature for 10 min (to reach 42 °C), wrapped in wrapping foil, and kept in a refrigerator at 4–8 °C for 24 h. Dough was mixed at medium speed in two consecutive 20 min steps. Yeast was activated in water for 10 min, at 35 °C, at 85% humidity (WIESHEU Wolfen GmbH, Bitterfeld-Wolfen, Germany) before addition to dough. At the beginning of the first phase of mixing, the dry floury ingredients (or sourdough) and water (Table 1) were mixed with ScD, while yeast was added 10 min after. Other ingredients were added to the dough at the beginning of the second phase of mixing, except salt, which was added two minutes before the end of mixing. Dough (100 g) was then transferred to aluminum molds (d = 13 cm) and fermented at 35 °C, at 85% relative humidity for one hour. Breads were baked in a deck oven (Ramalhos S.A., Águeda, Portugal), at 280 °C, for 3.5 min, flipped, and baked for an additional 2.5 min in the mold. Prior to further analysis, bread was cooled at room temperature for one hour on a porous pan. Bread samples were packed in PA/PE plastic bags and stored in a climate chamber (HPP110, Memmert GmbH, Schwabach, Germany) at 20 °C until further analysis.

### 2.5. Sourdough Fermentation

The CSPF_LF+KM bread was prepared using the optimized formulation and fermentation protocol previously described [9]. Briefly, sourdough was prepared according to Table 1, with a dough yield of 400 (flour-to-water ratio 1:3). The dough was inoculated with *Limosilactobacillus fermentum* (10^7^ CFU g^−1^) and *Kluyveromyces marxianus* (10^5^ CFU g^−1^) and fermented at 35 °C until pH 4.1 was reached after approximately 6.5 h.

### 2.6. Chemical Analyses of Bread

Chemical composition of baked breads was determined in duplicate according to standard methods: protein content by the AACC 46-12 Kjeldahl method using factor 6.25 for nitrogen-protein conversion; moisture content by the AACC 45-15.02 method; total fat content by the Soxhlet method AACC 30-23; ash content by AACC 08-01 method; sugar by Luff Schoorl method AACC 80-68 [22]. Additionally, soluble and insoluble fiber content was determined by the AOAC method 2009.01 and 2011.25 [23], using an enzymatic kit, integrated total dietary fiber assay kit (Megazyme, Wicklow, Ireland). Total carbohydrate content was calculated by subtraction of protein, fat, ash, fiber, and water content (g 100 g^−1^) from the total weight of the sample (100 g).

The concentrations of minerals in breads were determined using atomic absorption spectrometry (AAS). Ashing of a well-homogenized sample (5 g) was performed in a muffle furnace (KR-170, Heraeus, Hanau, Germany) at 550 °C according to the AOAC method 923.03 [23], as described in [24], and the concentrations were measured on the atomic absorption spectrometer (Perkin Elmer 2380; Norwalk, CT, USA).

Fructan content was determined using a Megazyme Fructan assay protocol (K-FRUC, Megazyme, Wicklow, Ireland), according to AOAC method 999.03 and AACC method 32-32.01 [22] with the use of Lambda 35, PerkinElmer, Überlingen, Germany.

### 2.7. Evaluation of Physical Properties of Bread

Physical properties of bread were determined one hour post-baking. Bread volume was determined as a minimum of five replicates by AACC Method 10-05.01 [22]. Specific volume was calculated as the volume-to-mass ratio. Bread dimensions were measured with a caliper (at min. 3 points), and spread was calculated as the width-to-height ratio. Baking loss was calculated according to the equation:

(1) $$Baking\;loss=\frac{m1-m2}{m1}\times 100$$

where

m_1_ is the mass of bread dough (g) prior to proofing.

m_2_ is the mass of bread (g) one hour post-baking.

Crust color was measured by the CIELab system color space values (L*, a*, and b*), using a Konica Minolta CM-700d colorimeter (Minolta, Tokyo, Japan) with the 7 mm slit cover. The browning index (BI) was calculated using the following equation:

(2) $$BI=\frac{100(x-0.31)}{0.17}\;\mathrm{where}\;x=\frac{a*+1.75L*}{5.64L*+a*-3.012b*}$$

### 2.8. Sensory Acceptability of Breads

Hedonic analysis was conducted in individual sensory booths according to ISO 6658:2017 [25] in the sensory laboratory at UNZG FFTB. Bread samples were evaluated 24 h after bread baking with 48 healthy participants (38 females and 10 males, age 26–64 years), employees and students of the UNZG FFTB. Participants were provided with water for palate cleansing between samples. The sensory study was approved by the UNZG FFTB, Zagreb, Croatia, on 8 July 2022. A 9-point hedonic scale was used by the assessors to indicate the extent to which they liked or disliked each bread sample. The scale ranged from 1 as ‘dislike extremely’ to 9 as ‘like extremely’.

### 2.9. Bread Storage Stability

The storage stability of GFFB was evaluated by microbiological analyses, together with texture profile analysis and sensory evaluation of bread staleness during 72 h.

#### 2.9.1. Microbiological Safety

Microbiological analyses of breads were limited to 72 h to ensure safety for human consumption during sensory evaluation. Total aerobic mesophilic bacteria were enumerated according to ISO 4833-1 [26] with the use of PCA plate, with plates incubated at 30 °C for 72 h. The presence of rope-forming spores was determined according to the AACC 42-20 method [22] with results recorded after 48 h of incubation at 35 °C. The enumeration of yeasts and molds was conducted according to ICC standard 146, with incubation at 25 °C for 72 h (yeasts) and 120 h (molds). To minimize post-baking contamination, surface decontamination of the samples, packaging, and laboratory work areas was performed using 96% (*v*/*v*) food-grade ethyl alcohol (Dana d.o.o., Mirna, Slovenia). The ethanol was applied uniformly by spraying both sides of the bread loaves and the inner surfaces of the storage bags. Following the treatment, the alcohol was allowed to evaporate completely, ensuring the samples were suitable for subsequent analyses.

#### 2.9.2. Texture Profile

Texture profile analysis (TPA) was determined on bread samples [27] at 2, 4, 20, 24, 48, and 72 h post-baking in at least six replicates. Hardness, resilience, cohesiveness, and chewiness were determined using a TA1 texture analyzer (Ametek Llyod Instruments Ltd., Bognor Regis, UK), fitted with an aluminum probe 55 mm in diameter and coupled with Nexygen PLUS 3 Software, at test speed 2 mm s^−1^, trigger force 5N, strain 50%, time between two compressions 30 s. Samples taken from the GF flatbread included both crumb and crust and consisted of two pieces (12.5 mm thick, 35 mm in diameter) placed on top of each other.

#### 2.9.3. Sensory Analysis of Bread Staleness

Sensory perception test of bread staleness was conducted according to AACC method 74-09 [22] and ISO 6658:2017 [25] in individual sensory booths in the sensory laboratory at UNZG FFTB. The sensory study was approved by the UNZG FFTB, Zagreb, Croatia, on 8 July 2022. The panel consisted of 16 healthy judges (13 female and 3 male, ages 26–60 years), employees of the UNZG FFTB with experience in performing sensory analyses of gluten-free bread. The panel members, with over 5 years of experience in gluten-free bread sensory analysis, participated in three calibration sessions prior to the study to standardize the evaluation of the freshness/staleness scale. Prior to the analysis, bread was cut into circles (diameter 35 mm). Panelists had to determine which term on a rating scale (very fresh—6, fresh—5, slightly fresh—4, slightly stale—3, stale—2, very stale—1) best represents the analyzed sample [22]. Participants were provided with water for palate cleansing between samples. The freshness of the bread was assessed according to feel in the finger, odor, flavor, and mouthfeel [22]. The evaluation was conducted over two sessions, where four samples of each bread type (aged 1, 24, 48, and 72 h) were presented simultaneously to the panelists.

### 2.10. Statistical Analysis

Statistical analysis was performed using Statistica 14.1.0 (TIBCO Software Inc., Palo Alto, CA, USA). Data were expressed as mean ± standard deviation. One-way ANOVA followed by Tukey’s post hoc test was used to determine differences among bread types at each storage time. Texture parameters were analyzed using two-way ANOVA with bread type and storage time as fixed factors. Two-way ANOVA was used to evaluate the main effects and their interaction, followed by Tukey’s post hoc test to compare bread types within each storage interval. Differences were considered significant at *p* < 0.05.

## 3. Results and Discussion

### 3.1. Pasting Properties

Since lower viscosity of gluten-free doughs may reduce their gas-holding capacity, which may result in lower bread volume [28], the viscosity of the individual flours and flour blends used in the preparation of GFFB (Table 1) was determined.

The pasting profile of the tested flours (Table 2) and flour blends used in the preparation of GFFB revealed pronounced differences in starch gelatinization behavior among the analyzed flours. RF exhibited the highest peak and final viscosities, indicating greater starch-swelling capacity and gel-forming potential, similar to millet flour, suggesting their suitability for gluten-free bakery applications, as a higher paste viscosity contributes to dough stabilization [28]. Both flours exhibited typical flour pasting behavior. WCF presented the lowest peak viscosity (Table 2), indicating limited swelling of starch granules and a weaker paste structure during cooling. The ScD showed a markedly different pasting profile due to the pre-gelatinization of starch during the scalding process, which explains the very low gelatinization onset temperature and peak viscosity. The scalding process may partially disrupt starch granules, limiting the ability of starch chains to reassociate during cooling and thereby reducing the formation of a stable gel network [29]. This effect is visible in the statistically significant decrease in setbact viscosity for ScD (118 BU) compared to WCF (223 BU).

The pasting properties of flour blends used in the preparation of bread formulations (CTRL-B, CSF-B, and CSPF-B) were analyzed to evaluate the synergistic effect of the ingredients on dough consistency. This approach was essential to assess the specific influence of CSF and CSPF, since their low starch content and high fiber or sugar content do not allow for the formation of a typical pasting curve independently. The addition of carob flours (CSF-B and CSPF-B) led to a slight but significant decrease in pasting temperature (73.7 °C and 73.4 °C, respectively) compared to CTRL-B (74.3 °C). Soluble dietary fiber may promote an earlier onset of viscosity due to its high water-binding and swelling properties, thereby modifying the rheological properties of the system and contributing to structural strengthening. CTRL-B and CSPF-B exhibited comparable pasting profiles, although CSPF-B reached a significantly lower peak viscosity (320 BU) than CTRL-B (397.5 BU). The higher viscosity of CTRL-B can be attributed to its greater proportion of RF, the component with the highest peak viscosity among the flours studied. The statistically higher setback viscosity in CTRL-B (571 BU) than in CSPF-B (366.5 BU) indicated more pronounced starch retrogradation of the control flour blend; this may be attributed to the fiber fraction in CSPF, which potentially hindered the tight realignment of starch chains. Also, the lower viscosity development of CSPF-B compared with CTRL-B may be explained by the high sugar content of carob pod flour (approximately 50%) [30], which reduces the relative starch content in the formulation and consequently its contribution to starch retrogradation. CSF-B exhibited an even more pronounced rise in viscosity compared to CTRL-B, reaching the highest peak viscosity among all flour blends (520 BU) and the largest setback viscosity (755 BU), which did not significantly differ from RF (770 BU) or PMF (734.5). Although setback viscosity is commonly associated with starch retrogradation, in this case, the pronounced increase during cooling may also reflect the contribution of soluble dietary fiber (Table 3) and galactomannans from carob seed flour [31], which may enhance water binding and structure formation in gluten-free dough [32].

### 3.2. Nutritive Value of Bread

The chemical composition and dietary fiber profile of the analyzed bread formulations are presented in Table 3, while the mineral composition is shown in Table 4. While the protein content remained comparable among formulations and only minor differences were observed in fat content, the incorporation of carob ingredients together with millet bran and millet flour resulted in noticeable changes in the dietary fiber composition compared to the control formulation. In particular, breads in which 10% of rice and corn flour was replaced with carob and millet ingredients (CSF-B and CSPF-B, Table 1) showed substantially higher dietary fiber levels (Table 3), reaching 8.2 and 8.3 g per 100 g of bread, respectively. Insoluble dietary fiber increased almost twofold in CSF-B and CSPF-B. Since similar increases were observed in both carob-containing breads, this effect can be attributed to the naturally high fiber content of millet bran. Analysis showed that dietary fiber accounted for approximately 37% of millet bran (0.37 g g^−1^ d.w.), with insoluble dietary fiber representing the predominant fraction (0.33 g g^−1^ d.w.). A pronounced increase was also observed for dietary fiber soluble in water and 78% aqueous ethanol (SDFS), as well as in water-soluble dietary fiber that precipitates in 78% aqueous ethanol (SDFP), which was more than doubled in CSF-B compared to CTRL-B and CSPF-B, reflecting the nutritional differences between CSF and CSPF, the former being rich in soluble dietary fiber. Increased dietary fiber content is particularly relevant for gluten-free products, which are often characterized by lower fiber levels compared to conventional wheat-based products [4]. In addition, carob seed fiber has been associated with several health benefits, including the stabilization of postprandial glucose levels and prebiotic effects [12,13]. Moreover, the higher fiber content, particularly soluble fractions, may also contribute to the water-binding and thickening behavior observed in the pasting profile of the batter. Compared with CSPF-B, sourdough fermentation resulted in a reduction in water-soluble dietary fiber that precipitates in 78% aqueous ethanol (SDFS) by approximately 59% and SDFP by about 61% in CSPF_LF+KM, which may reflect enzymatic conversions and microbial metabolism of fermentable carbohydrates [13].

Considering the relatively high carbohydrate content of carob pods [30], the determination of fructans, one of the main FODMAP compounds (fermentable oligo-, di- and monosaccharides and polyols), was therefore included in the analysis. FODMAPs may exert a prebiotic effect in healthy individuals but may cause gastrointestinal symptoms in sensitive individuals [33]. The fructan content in CTRL-B was 0.05 g 100 g^−1^. Enrichment with CSF and CSPF increased the fructan content to 0.07 and 0.09 g 100 g^−1^, respectively. Sourdough fermentation of CSPF reduced the fructan level to 0.03 g 100 g^−1^, consistent with previous findings showing that bioprocessing can effectively reduce fructan content [34,35]. Notably, the fructan content in all bread samples remained below the threshold of 0.5 g per serving considered potentially problematic for sensitive individuals [36].

A similar trend was observed for mineral composition. In particular, iron content in CSF-B was almost two-fold higher than in the control formulation and approximately 36% higher than in CSPF-B (Table 4). Higher iron concentrations in carob seeds compared to pods were previously reported [37], and is consistent with the higher iron content observed in CSF-B. Breads containing carob ingredients also exhibited higher ash content, suggesting the mineral contribution of both carob and millet ingredients. Nevertheless, the higher ash content of CSPF-B suggests that CSPF may contribute more to the overall mineral content. Overall, the incorporation of carob and millet ingredients improved the nutritional profile of GFFB, primarily through increased dietary fiber and mineral content.

### 3.3. Physical Properties of Bread

Physical properties of the analyzed breads indicate improved technological performance with the incorporation of CSF. CSF-B exhibited lower baking loss compared to the other formulations, with values similar to those (20.9) previously reported for CSPF_LF+KM bread [9]. This result is consistent with its higher moisture content (Table 3) and the increased dietary fiber content introduced by CSF, which enhances water binding within the dough.

The higher specific volume obtained in CSF-B compared to CTRL-B may be explained by the pasting characteristics of the flour mixture (Table 2). The higher peak and final viscosity in the presence of CSF allows a longer time for water absorption during heating, which may contribute to improved dough expansion and consequently a higher volume of the final product. The specific volume achieved in CSF-B was comparable to that reported for the CSPF_LF+KM sourdough bread. For a comprehensive comparison, the physical properties of the CSPF_LF+KM sample, previously reported in [9], were included in the statistical analysis and Table 5. The lower diameter-to-height ratio observed for CSF-B, similar to CSPF_LF+KM (13.3), indicates improved structural stability during baking, which may be attributed to the presence of dietary fiber acting as a hydrocolloid. Carob-derived gum has been reported to retain water and form a gelling network during proofing and baking, contributing to the elasticity and structural symmetry of gluten-free bread [15] in CSF-B and by the sourdough fermentation process in CSPF_LF+KM. The addition of carob ingredients and PMB led to a significant decrease in L* values, resulting in a darker crumb colour. Additionally, sourdough fermentation significantly intensified crumb color in CSPF_LF+KM bread, as the browning index of CSPF-B was approximately 20% lower than that reported for CSPF_LF+KM (6.73). This indicates that sourdough fermentation contributed substantially to color development, as it increases the availability of precursors that participate in Maillard reactions during baking [9,38]. Nevertheless, all bread samples exhibited acceptable physical quality parameters, indicating that the incorporation of carob ingredients did not adversely affect the technological performance of the gluten-free bread formulations. Both sourdough fermentation of CSPF and the use of CSF showed similarly positive effects, supporting their role in improving the structural stability of gluten-free dough.

### 3.4. Sensory Acceptability of Breads

The results of the hedonic sensory evaluation are presented in Table 6. No statistically significant differences (*p* > 0.05) were observed among the bread samples for appearance, odor, flavor, taste, texture, or overall impression. These results indicate that the incorporation of CSF, CSPF, as well as sourdough CSPF_LF+KM, did not negatively affect consumer acceptance of the breads compared to the control formulation. All samples received comparable scores, with mean values ranging between 6.50 and 6.79 for overall acceptability, indicating slight to moderate liking of GFFB and potential for further development.

### 3.5. Bread Storage Stability

#### 3.5.1. Microbiological Safety

Microbiological safety of the breads was monitored during 72 h of storage by enumeration of total aerobic mesophilic bacteria, rope-forming spores, yeast, and molds. In all samples, the total aerobic mesophilic count remained below 10^6^ CFU g^−1^ throughout the storage period, the yeast count remained below 10 CFU g^−1^, and no mold growth was observed (<10 CFU g^−1^). The enumeration of rope-forming bacteria also confirmed the absence of rope spoilage. These results indicate that all bread formulations maintained satisfactory microbiological quality for 72 h after baking.

#### 3.5.2. Texture Profile

**Figure 1:**
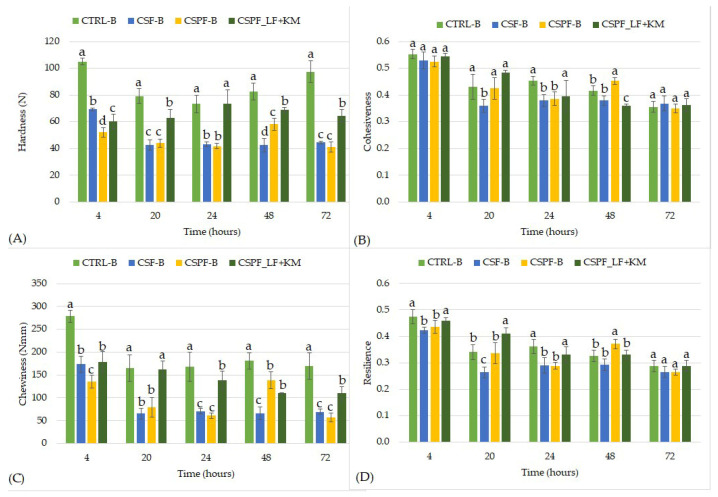
Changes in textural properties of bread crumb samples during 72 h of storage: (**A**) hardness, (**B**) cohesiveness, (**C**) chewiness, and (**D**) resilience. Different letters within the same storage time indicate statistically significant differences among samples (*p* < 0.05). Values are presented as mean ± standard deviation (*n* ≥ 6). Abbreviations: CTRL-B, control bread (rice flour and scalded white corn flour); CSF-B, bread containing carob seed flour; CSPF-B, bread containing carob seed and pod flour; CSPF_LF+KM, bread containing fermented carob seed and pod flour.

The TPA enables comparison of different formulations at the fresh stage and during storage. Two hours post-baking, hardness and chewiness showed the largest differences among the tested samples. CSF-B showed significantly lower initial hardness (59.3 N) compared to CTRL-B (116.8 N) and CSPF-B (82.4 N) (Figure 1). Once again, the impact of adding CSF was comparable to that of sourdough fermentation, as CSPF_LF+KM exhibited a hardness value of 52.8 N [9]. Similarly, the initial chewiness of CSF-B (148.8 N·mm) was significantly lower than that of CSPF-B, which showed values comparable to its fermented counterpart CSPF_LF+KM (186 N·mm) [9], and approximately 50% lower than that of CTRL-B (299.8 N·mm). These results suggest that the incorporation of CSF contributes to a softer crumb structure of a freshly baked GFFB, likely associated with improved water retention (Table 3).

Two-way ANOVA revealed significant effects of bread type, storage time, and their interaction for all texture parameters (*p* < 0.0001, Table S1, Supplementary Materials). Sourdough fermentation was treated as part of the bread formulation factor. Specifically, for hardness, bread formulation accounted for the largest proportion of total variance (58.4%, F(3, 119) = 478, *p* < 0.0001). Along with its significant interaction with storage time (19.1% of variance, F(15, 119) = 31.3, *p* < 0.0001), formulation was the key determinant of crumb hardening. This observation is consistent with previous studies reporting a strong influence of flour type on the textural properties of gluten-free bread [39]. Indeed, hardness values differed markedly among formulations and throughout storage.

While the sourdough bread CSPF_LF+KM, which had a lower initial hardness, displayed a generally expected staling behavior and an increase in hardness of approximately 22% during storage, CTRL-B showed a decrease in hardness until 20 h of storage, followed by a gradual increase, with the final hardness still remaining below the initial value. Such changes may be associated with moisture redistribution within the bread matrix. After baking, a moisture gradient exists between the crust and crumb, and changes in crumb hardness may occur as a result of moisture redistribution during storage [40,41]. CTRL-B also exhibited the highest initial hardness and maintained significantly higher values compared to the composite formulations during storage. In contrast, CSF-B and CSPF-B exhibited substantially lower hardness values and a less pronounced firming trend during storage. The slower bread hardening observed in CSF-B and CSB-B indicates that the dietary fiber content in these breads may contribute to delayed bread staling. This observation is consistent with the pasting behavior of the flour blends (Table 2), since formulations containing higher proportions of RF and ScD (CTRL-B) provide more starch available for retrogradation, whereas carob-containing breads have a lower relative starch content [30] and higher levels of water-binding dietary fiber (Table 3), which may limit starch reassociation and slow crumb firming during storage.

In contrast to the ANOVA results for hardness, cohesiveness and resilience were mainly influenced by storage time, explaining 69.4% and 63.8% of total variation, respectively, (F(5, 119)= 175; F(5, 116) = 183, *p* < 0.0001), wheras the effect of bread type remained limited (3.69%, F(3, 119) = 15.5 and 8.7%, F(5, 116) = 41.5, respectively). Changes in crumb structural properties primarily driven by processes occurring during storage are commonly associated with starch retrogradation [42]. For these parameters, CSPF_LF+KM showed the highest values, comparable to CTRL-B during the first 20 h of storage, after which they declined to levels similar to CSPF-B, while CSF-B exhibited the lowest values throughout the 72 h storage period. The lower cohesiveness and resilience observed for CSF-B may reflect the higher fiber content of the formulation, as dietary fibers exhibit high water-holding capacity and can modify the technological properties of cereal-based systems [43]. Replacement of starch with citrus fiber has been shown to decrease bread cohesiveness and alter crumb characteristics [28]. Resilience quantifies the instant recovery capacity of the crumb after compression and therefore reflects the ability of the structure to regain its original form [44]. The lower resilience values observed in these samples may therefore originate from the same structural characteristic responsible for the lower hardness, namely a more porous crumb structure that undergoes greater deformation during compression. Nevertheless, CSF-B exhibited the lowest decline through 72 h in cohesiveness and resilience during storage, indicating improved stability of crumb structure compared to the control formulation. Despite significant differences in nutritional composition and water-binding capacity (Table 3), CSF-B and CSPF-B exhibited similar textural behavior during the 72 h storage period. This suggests that ingredients shared by both CSF-B and CSPF-B formulations, particularly PMF and PMB, may also contribute to their similar textural behavior during storage.

#### 3.5.3. Sensory Analysis of Bread Staleness

Sensory analysis of perceived freshness during 72 h of storage was performed in order to complement the textural evaluation of bread. Bread freshness perception is a complex sensory phenomenon involving interactions between appearance, odor, taste, flavor, and oral texture attributes [45].

**Figure 2:**
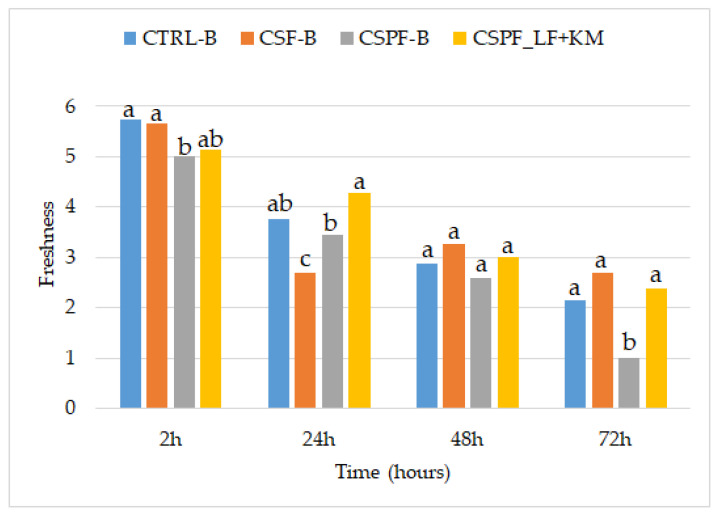
Results of sensory evaluation of bread freshness during 72 h of storage. Different letters within the same storage time indicate statistically significant differences among samples (one-way ANOVA followed by Tukey’s HSD test, *p* < 0.05). Abbreviations: CTRL-B, control bread (rice flour and scalded white corn flour); CSF-B, bread containing carob seed flour; CSPF-B, bread containing carob seed and pod flour; CSPF_LF+KM, bread containing fermented carob seed and pod flour.

The perceived freshness of these GFFB is a complex attribute influenced by the interplay of moisture retention, fiber content, and sourdough acidification. The CSPF-B showed significantly lower freshness perception already at the initial testing compared to the control and CSF-B. A similar pattern of statistically significant differences was observed after 72 h of storage, with CSPF-B again being the only sample that significantly deviated due to its lower freshness perception (Figure 2). In addition, CSPF-B exhibited the greatest decline in perceived freshness during storage, which may be associated with the crumbly texture observed by the sensory panel after 72 h. This structural instability in CSPF-B may be caused by the specific fiber content of the carob pod, disrupting the starch matrix. In contrast, CSPF_LF+KM was perceived as the freshest sample after 24 h and exhibited the smallest overall decrease in freshness during the 72 h storage period. This improvement may be attributed to the sourdough acidification, which has been shown to improve the technological properties of bread, resulting in a softer crumb [46]. Acidification may activate endogenous enzymes, such as proteases and amylases, which may modify starch and protein matrix, thereby reducing crumbliness [47]. Significant reduction in soluble fiber fractions observed in CSPF_LF+KM (Table 3) likely diminished the interference of the pod-derived components with the starch gel, resulting in a more cohesive crumb structure. Nevertheless, after 48 h of storage, all samples were perceived as slightly stale, indicating a general decline in sensory freshness. Overall, these results highlight the importance of sensory evaluation in GFFB storage stability assessment, as textural and microbiological analyses alone were not sufficient to fully reflect changes in perceived freshness.

Limitations of the Study: Despite the promising results regarding the use of carob flour and sourdough, some limitations should be noted. In order to provide a more comprehensive understanding of moisture migration and microbial stability, and further support the validation of these enriched gluten-free formulations, future research should include the determination of water activity (aw) of the bread samples during storage, as well as additional microbiological indicators such as *Enterobacteriaceae* and coliforms.

## 4. Conclusions

This study demonstrated that carob seed flour, as well as whole carob fruit flour together with sourdough fermentation, can serve as effective clean-label strategies for improving the nutritional and technological quality, as well as perception of freshness of gluten-free flatbread throughout 72 h, without changing their sensory acceptability. Both whole carob fruit flour and carob seed flour contributed to the content of dietary fiber of the breads; however, carob seed flour showed the most favorable technological performance, mainly through improved batter rheology, higher specific volume (23%), reduced baking loss (8%), and a softer crumb structure (50% lower hardness) compared to control, as well as maintaining a softer bread structure throughout 72 h of storage. In contrast, breads containing flour obtained from the whole carob fruit initially exhibited weaker technological performance. However, sourdough fermentation effectively compensated for the initially weaker technological performance of whole carob fruit formulations, achieving textural properties comparable to those obtained with carob seed flour. The addition of carob flours or sourdough did not significantly alter the hedonic sensory aspects (appearance, odor, flavor, and overall impression) compared to the control, confirming high consumer acceptance. Overall, the results highlight the importance of sensory evaluation in bread storage stability assessment, as it revealed the significant improvement of freshness perception with sourdough fermentation. However, the storage stability assessment was limited to a 72 h period, during which all samples followed a typical trend of decline in sensory freshness. Future research should explore the fermentation of carob seed flour to further investigate its potential as a natural improver for the storage stability of gluten-free bakery products.

## Figures and Tables

**Table 1:** Formulations of bread samples.

Ingredient (%)	CTRL-B	CSF-B	CSPF-B	CSPF_LF+KM
ScD (of which WCF)	105 (50)	94.5 (45)	94.5 (45)	94.5 (45)
RF	50	45	45	45
PMB	-	5.25	5.25	5.25 *
PMF	-	2.17	2.17	2.17 *
CSPF	-	-	2.58	2.58 *
CSF	-	2.58	-	-
Tap water	110	115	115	115
All recipes contained 0.66% sodium bicarbonate, 1% salt, 1% oil, 1.3% yeast, 5% sugar, based on the total flour weight.				

* Ingredients were fermented into sourdough according to the procedure described in 2.5. Percentages are expressed on a flour basis, calculated relative to the total amount of flour ingredients. Abbreviations: RF, whole rice flour; WCF, white corn flour; ScD, scalded white corn flour; PMF, proso millet flour; PMB, proso millet bran; CSPF, carob seed and pod flour; CSF, carob seed flour; CTRL-B, control bread (rice flour and scalded white corn flour); CSF-B, bread containing carob seed flour; CSPF-B, bread containing carob seed and pod flour.

**Table 2:** Pasting properties of flours and flour blends used in the preparation of GFFB.

Sample	Gelatinization Onset Temperature (°C)	Peak Viscosity (BU)	Final Viscosity (BU)	Setback (BU)
RF	79.0 ± 0.7 ^b^	701.5 ± 4.9 ^a^	1471.5 ± 9.2 ^a^	770.0 ± 4.2 ^a^
PMF	82.5 ± 0.7 ^a^	561.5 ± 4.9 ^b^	1296.0 ± 31.1 ^b^	734.5 ± 26.2 ^a^
WCF	64.0 ± 0.7 ^e^	258.5 ± 9.2 ^f^	481.5 ± 4.9 ^e^	223.0 ± 4.2 ^d^
ScD	31.5 ± 0.7 ^f^	163.5 ± 12.0 ^g^	281.5 ± 19.1 ^f^	118.0 ± 7.1 ^e^
CTRL-B	74.3 ± 0.1 ^c^	397.5 ± 3.5 ^d^	968.5 ± 9.2 ^c^	571.0 ± 5.7 ^b^
CSF-B	73.7 ± 0.2 ^cd^	520.0 ± 14.1 ^c^	1275.0 ± 38.2 ^b^	755.0 ± 24.0 ^a^
CSPF-B	73.4 ± 0.2 ^d^	320.0 ± 21.2 ^e^	686.5 ± 40.3 ^d^	366.5 ± 19.1 ^c^

Values are expressed as mean ± standard deviation (*n* ≥ 2). Different superscript letters within the same column indicate statistically significant differences among samples (*p* < 0.05). Abbreviations: RF, whole rice flour; PMF, proso millet flour; WCF, white corn flour; ScD, scalded white corn flour; CTRL-B, flour mixture used in preparation of control bread; CSF-B, flour mixture used in preparation of bread containing carob seed flour; CSPF-B, flour mixture used in preparation of bread containing carob seed and pod flour; BU, Brabender units.

**Table 3:** Chemical composition of bread formulations.

Component (g 100 g^−1^ d.w.)	CTRL-B	CSF-B	CSPF-B	CSPF_LF+KM
Fat	2.37 ± 0.06 ^a^	2.07 ± 0.00 ^b^	2.10 ± 0.05 ^b^	2.09 ± 0.05 ^b^
Minerals as ash	2.39 ± 0.00 ^b^	2.51 ± 0.06 ^ab^	2.70 ± 0.11 ^a^	2.68 ± 0.03 ^a^
Protein	9.14 ± 0.01 ^a^	9.37 ± 0.12 ^a^	9.47 ± 0.07 ^a^	9.53 ± 0.22 ^a^
IDF	2.89 ± 0.08 ^b^	5.10 ± 0.19 ^a^	5.40 ± 0.76 ^a^	5.44 ± 0.08 ^a^
SDFP	0.80 ± 0.08 ^c^	2.39 ± 0.05 ^a^	1.21 ± 0.26 ^b^	0.49 ± 0.10 ^d^
SDFS	4.12 ± 0.40 ^b^	5.20 ± 0.03 ^a^	5.34 ± 0.04 ^a^	2.06 ± 0.00 ^c^
Carbohydrates	48.39	37.6	43.28	47.38
Moisture (%)	29.9 ± 0.4 ^b^	35.8 ± 0.5 ^a^	30.5 ± 0.3 ^b^	30.3 ± 0.3 ^b^

Values are expressed as mean ± standard deviation (*n* ≥ 2). Different superscript letters within the same row indicate statistically significant differences among samples (*p* < 0.05). Abbreviations: CTRL-B, control bread (rice flour and scalded white corn flour); CSF-B, bread containing carob seed flour; CSPF-B, bread containing carob seed and pod flour; IDF, insoluble dietary fiber; SDFP, water-soluble dietary fiber that precipitates in 78% aqueous ethanol; SDFS, dietary fiber soluble in water and 78% aqueous ethanol; d.w., dry weight.

**Table 4:** Mineral composition of bread samples expressed on a dry-weight basis.

Bread Formulation	Cu (μg g^−1^ d.w.)	Fe (μg g^−1^ d.w.)	Zn (μg g^−1^ d.w.)
CTRL-B	1.90 ± 0.03 ^b^	11.56 ± 2.24 ^c^	13.99 ± 1.46 ^a^
CSF-B	2.05 ± 0.06 ^a^	21.75 ± 3.32 ^a^	12.48 ± 0.10 ^b^
CSPF-B	1.45 ± 0.29 ^c^	15.97 ± 0.34 ^b^	12.60 ± 0.40 ^b^
CSPF_LF+KM	1.38 ± 0.40 ^c^	15.85 ± 0.9 ^b^	13.06 ± 0.55 ^ab^

Values are presented as mean ± standard deviation (*n* ≥ 3). Different superscript letters within the same row indicate statistically significant differences among samples (*p* < 0.05). Abbreviations: CTRL-B, control bread (rice flour and scalded white corn flour); CSF-B, bread containing carob seed flour; CSPF-B, bread containing carob seed and pod flour; d.w., dry weight.

**Table 5:** Physical properties of bread.

	CTRL-B	CSF-B	CSPF-B	CSPF_LF+KM
	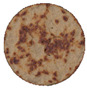	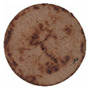	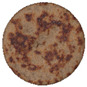	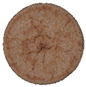
Baking loss%	22.0 ± 0.7 ^a^	20.6 ± 0.8 ^b^	22.3 ± 0.1 ^a^	20.9 ± 0.9 ^ab^
Specific volume (mL g^−1^)	1.7 ± 0.1 ^c^	2.1 ± 0.1 ^a^	1.8 ± 0.1 ^b^	2.0 ± 0.05 ^a^
Spread (d/h)	14.3 ± 0.1 ^a^	13.8 ± 0.1 ^b^	14.2 ± 0.1 ^a^	13.3 ± 0.8 ^c^
L*	54.3 ± 0.1 ^a^	51.5 ± 0.7 ^b^	52.1 ± 0.8 ^b^	52.3 ± 0.6 ^b^
a*	1.77 ± 0.5 ^a^	0.9 ± 0.4 ^b^	1.3± 0.5 ^ab^	1.2 ± 0.2 ^ab^
b*	1.1 ± 0.3 ^a^	1.8 ± 0.7 ^ab^	1.9 ± 0.5 ^ab^	2.6 ± 0.6 ^b^
Browning index	4.5	4.7	5.4	6.7

Values are expressed as mean ± standard deviation (*n* ≥ 5). Different letters within the same row indicate statistically significant differences among samples (*p* < 0.05). Abbreviations: CTRL-B, control bread (rice flour and scalded white corn flour); CSF-B, bread containing carob seed flour; CSPF-B, bread containing carob seed and pod flour. Values for CSPF_LF+KM are adapted from [9] and included for comparative statistical analysis.

**Table 6:** Hedonic sensory evaluation scores of bread samples were evaluated 24 h after baking.

Sample	Appearance	Odor	Flavor and Taste	Texture	Overall Impression
CSF-B	6.74 ± 1.81	7.00 ± 1.81	6.55 ± 1.73	6.60 ± 1.74	6.64 ± 1.65
CSPF-B	6.25 ± 2.09	6.83 ± 1.63	6.67 ± 1.66	5.96 ± 1.83	6.50 ± 1.64
CTRL-B	6.79 ± 1.79	7.17 ± 1.52	6.96 ± 1.60	6.75 ± 1.62	6.79 ± 1.54
CSPF_LF+KM	6.54 ± 1.93	6.63 ± 1.71	6.71 ± 1.23	6.92 ± 1.28	6.54 ± 1.56

Values are expressed as mean ± standard deviation (SD). Abbreviations: CTRL-B, control bread (rice flour and scalded white corn flour); CSF-B, bread containing carob seed flour; CSPF-B, bread containing carob seed and pod flour; CSPF_LF+KM, bread containing fermented carob seed and pod flour. No statistically significant differences (*p* > 0.05) were found among the evaluated samples for appearance, odor, flavor, aroma, texture, and overall acceptability.

## Data Availability

The original contributions presented in this study are included in the article/Supplementary Material. Further inquiries can be directed to the corresponding author.

## Supplementary Materials

The following supporting information can be downloaded at https://www.mdpi.com/article/10.3390/foods15091504/s1. Table S1: Two-way ANOVA summary for texture parameters (hardness, cohesiveness, resilience, and chewiness) with bread type and storage time as fixed factors.

## Abbreviations

The following abbreviations are used in this manuscript:

GFFB	Gluten-free flatbread
RF	Whole-grain rice flour
PMF	Proso millet flour
PMB	Proso millet bran
WCF	White corn flour
ScD	Scalded white corn flour
CTRL-B	Control bread containing rice and corn flour
CSF	Carob seed flour
CSPF	Carob seed and pod flour
CSF-B	Bread containing carob seed flour
CSPF-B	Bread containing carob seed and pod flour
CSPF_LF+KM	Bread containing fermented carob seed and pod flour
UNZG FFTB	University of Zagreb, Faculty of Food Technology and Biotechnology
FODMAP	Fermentable oligo-, di- and monosaccharides and polyols
IDF	Insoluble dietary fiber
SDFP	Water-soluble dietary fiber that precipitates in 78% aqueous ethanol
SDFS	Dietary fiber soluble in water and 78% aqueous ethanol
BU	Brabender units
