# Correction: Voučko et al. Gluten-Free Flatbread with Carob Flour and Sourdough: Nutritional Composition, Technological Properties and Storage Stability. *Foods* 2026, *15*, 1504

**DOI:** 10.3390/foods15111963

**Published:** 2026-06-02

**Authors:** Bojana Voučko, Saša Drakula, Nikolina Čukelj Mustač, Vedrana Pleš, Ljiljana Nanjara, Tomislava Grgić, Dubravka Novotni

**Affiliations:** 1 Department of Food Engineering, Faculty of Food Technology and Biotechnology, University of Zagreb, 10 000 Zagreb, Croatia; 2 Department of Food Processing Technology, Karlovac University of Applied Sciences, 47 000 Karlovac, Croatia; 3 Department of Food Technology, University of Applied Sciences “Marko Marulić”, 22 300 Knin, Croatia

## 1. Error in Table

In the original publication [1], there was a mistake in Table 3 due to an incorrect dilution factor and dietary fiber calculations, affecting the IDF, SDFS and Carbohydrate values. Additionally, the rows for SDFP and SDFS were inadvertently interchanged. The corrected Table 3 appears below.

Furthermore, in Table 4, due to a typographical error during the final table formatting, the significance letters for zinc (Zn) were incorrectly presented as a, d, c, b instead of a, b, b, ab. The mean values and standard deviations for Zn remained correct.

## 2. Text Correction

Due to the updated calculations in Table 3, the value of dietary fiber increase in the Abstract section was updated from “nearly doubling” to “by over 60%” and the revised sentence should read as follows: “The incorporation of carob and millet ingredients improved the nutritional profile of GFFB, increasing total dietary fiber by over 60% and nearly doubling the iron content, without compromising sensory acceptance.”

To ensure consistency with the updated results in Table 3, a correction has been made to the Discussion, Section 3, Section 3.2. The fiber content for the fresh bread sample was updated from 10.4 g/100 g to 8.2 g/100 g, and the Discussion was modified to accurately reflect these results. The revised text should read as follows: “In particular, breads in which 10% of rice and corn flour was replaced with carob and millet ingredients (CSF-B and CSPF-B, Table 1) showed substantially higher dietary fiber levels (Table 3), reaching 8.2 and 8.3 g per 100 g of bread, respectively.”

In Section 3.2, part of a sentence was added to better reflect the change in fiber content, consistent with the changes in Table 3: “A pronounced increase was also observed for dietary fiber soluble in water and 78% aqueous ethanol (SDFS), as well as in water-soluble dietary fiber that precipitates in 78% aqueous ethanol (SDFP), which was more than doubled in CSF-B compared to CTRL-B and CSPF-B, reflecting the nutritional differences between CSF and CSPF, the former being rich in soluble dietary fiber.” 

The authors state that the scientific conclusions are unaffected. This correction was approved by the Academic Editor. The original publication has also been updated.

## Figures and Tables

**Table 3:** Chemical composition of bread formulations.

Component (g 100 g^−1^ d.w.)	CTRL-B	CSF-B	CSPF-B	CSPF_LF+KM
Fat	2.37 ± 0.06 ^a^	2.07 ± 0.00 ^b^	2.10 ± 0.05 ^b^	2.09 ± 0.05 ^b^
Minerals as ash	2.39 ± 0.00 ^b^	2.51 ± 0.06 ^ab^	2.70 ± 0.11 ^a^	2.68 ± 0.03 ^a^
Protein	9.14 ± 0.01 ^a^	9.37 ± 0.12 ^a^	9.47 ± 0.07 ^a^	9.53 ± 0.22 ^a^
IDF	2.89 ± 0.08 ^b^	5.10 ± 0.19 ^a^	5.40 ± 0.76 ^a^	5.44 ± 0.08 ^a^
SDFP	0.80 ± 0.08 ^c^	2.39 ± 0.05 ^a^	1.21 ± 0.26 ^b^	0.49 ± 0.10 ^d^
SDFS	4.12 ± 0.40 ^b^	5.20 ± 0.03 ^a^	5.34 ± 0.04 ^a^	2.06 ± 0.00 ^c^
Carbohydrates	48.39	37.6	43.28	47.38
Moisture (%)	29.9 ± 0.4 ^b^	35.8 ± 0.5 ^a^	30.5 ± 0.3 ^b^	30.3 ± 0.3 ^b^

Values are expressed as mean ± standard deviation (*n* ≥ 2). Different superscript letters within the same row indicate statistically significant differences among samples (*p* < 0.05). Abbreviations: CTRL-B, control bread (rice flour and scalded white corn flour); CSF-B, bread containing carob seed flour; CSPF-B, bread containing carob seed and pod flour; IDF, insoluble dietary fiber; SDFP, water-soluble dietary fiber that precipitates in 78% aqueous ethanol; SDFS, dietary fiber soluble in water and 78% aqueous ethanol; d.w., dry weight.
