# Supplementary material for: Gluten-Free Flatbread with Carob Flour and Sourdough: Nutritional Composition, Technological Properties and Storage Stability
Source: Foods. 2026 Apr 25;15(9):1504. doi: 10.3390/foods15091504 (PMC13163807; doi:10.3390/foods15091504)
Supplement: Supplementary file 1 [file foods-15-01504-s001.zip › foods-4250417-supplementary.pdf]

Table S1. Two-way ANOVA summary for texture parameters (hardness, cohesiveness, resilience, and chewiness) with bread type and storage time as fixed factors

Parameter	Source of Variation	% of total variation	F-value	DF	P value
Hardness	Interaction	19.1	31.3	15	< 0.0001
	Bread Type	58.4	478	3	< 0.0001
	Storage Time	12.1	59.3	5	< 0.0001
	Residual	10.4	-	119	-
Cohesiveness	Interaction	9.3	7.8	15	< 0.0001
	Bread Type	3.69	15.5	3	< 0.0001
	Storage Time	69.4	175	5	< 0.0001
	Residual	17.61	-	119	-
Resilience	Interaction	7.8	7.4	15	< 0.0001
	Bread Type	8.7	41.5	3	< 0.0001
	Storage Time	63.8	183	5	< 0.0001
	Residual	19.7	-	116	-
Chewiness	Interaction	8.9	11.6	15	< 0.0001
	Bread Type	39.6	259	3	< 0.0001
	Storage Time	35.7	140	5	< 0.0001
	Residual	15.8	-	119	-
